# Health services in older psoriasis patients before and after nursing home admission: A retrospective analysis of health insurance data

**DOI:** 10.1007/s00391-022-02020-y

**Published:** 2022-03-21

**Authors:** Jana Petersen, Claudia Garbe, Sandra Wolf, Brigitte Stephan, Matthias Augustin, Kristina Hagenström

**Affiliations:** Institute for Health Services Research in Dermatology and Nursing (IVDP), University Medical Center Hamburg-Eppendorf (UKE), Martinistr. 52, 20246 Hamburg, Germany

**Keywords:** Healthservices research, Elderly, Germany, Claims data, Nursing home residents, Versorgungsforschung, Senioren, Deutschland, Krankenkassendaten, Pflegeheimbewohner

## Abstract

**Background and objective:**

Demographic change confers significance to healthcare management of chronic diseases like psoriasis. There are few studies on the care of older people with psoriasis, particularly for the nursing home setting. It was investigated whether the number of psoriasis patients with specialist contact changes before vs. after nursing home admission.

**Material and methods:**

We analyzed claims data of a German health insurance company including a cohort of newly admitted nursing home residents aged 65 years and older between 2011 and 2014, who received a diagnosis of psoriasis 1 year before nursing home admission. Outpatient care was compared between the years before vs. after nursing home admission. We conducted a multivariate regression analysis for identifying predictors for dermatological care.

**Results:**

The study cohort included 718 insured persons (Ø83 years). Proportion of patients who had contact to a dermatologist significantly decreased after nursing home entry (44.6% before vs. 40.1% after nursing home entry). Strongest predictors for dermatological care after entry were a previously existing dermatological contact (odds ratio, OR 3.87, 95% confidence interval, CI 2.70–5.54) and prescription for topical steroids (OR 1.61, 95% CI 1.14–2.28).

**Conclusion:**

The analysis of health insurance data showed a pertinent decrease in the use of outpatient dermatological care after institutionalization. The evaluation of the adequacy of care is difficult due to the used database without clinical information. As long as no further investigations of this vulnerable patient group are available, the care of psoriasis patients of old age should be closely monitored. Dermatological knowledge of the skin in old age is an essential prerequisite for this.

**Supplementary Information:**

The online version of this article (10.1007/s00391-022-02020-y) contains supplementary material, which is available to authorized users.

## Abstract (de)

**Hintergrund und Fragestellung:**

National wie auch international gibt es nur wenige Informationen zur Versorgungssituation von älteren Menschen mit Psoriasis. Dies gilt im Besonderen für das Setting Pflegeheim. Wir untersuchten daher, inwieweit sich die ärztliche Versorgung durch den Eintritt in ein Pflegeheim verändert hat.

**Material und Methoden:**

Wir analysierten Abrechnungsdaten einer deutschlandweit tätigen Krankenkasse, der DAK-Gesundheit. Es wurden Versicherte untersucht, die in den Jahren 2011 bis 2014 neu in ein Pflegeheim aufgenommen wurden, mindestens 65 Jahre alt waren und im Jahr vor Heimeintritt mindestens eine gesicherte ambulant-ärztliche oder stationäre Psoriasis-Diagnose erhielten. Die ambulant-ärztliche Versorgung wurde im Jahr vor bzw. nach dem Eintritt ins Pflegeheim betrachtet. Anhand einer multivariaten Regressionsanalyse wurden Prädiktoren für eine dermatologische Versorgung identifiziert.

**Ergebnisse:**

Es konnten 718 Pflegeheimbewohner (Durchschnittsalter: 83 Jahre), die prävalent an einer Psoriasis erkrankt sind, in die Untersuchung miteinbezogen und ein Jahr vor bzw. nach Heimeintritt untersucht werden. Die Analyse der fachärztlichen Versorgung zeigt eine Reduktion des dermatologischen Kontakts nach Heimeintritt (45 % vs. 40 %). Als wichtigste Prädiktoren für eine dermatologische Versorgung nach Heimeintritt sind die dermatologische Versorgung vor Eintritt in ein Pflegeheim sowie die Verordnung topischer Steroide.

**Schlussfolgerung:**

Die Analyse der Krankenkassendaten zeigt einen deutlichen Rückgang der Inanspruchnahme ambulanter dermatologischer Versorgung nach der Heimunterbringung. Die Bewertung der Angemessenheit der Versorgung ist aufgrund der genutzten Datenbasis schwierig. Solange keine weiterführenden Untersuchungen dieser vulnerablen Patientengruppe vorliegen, gilt es, die Versorgung Psoriasiserkrankter aufmerksam zu beobachten. Dermatologisches Wissen von allen an der Versorgung Beteiligten über die Versorgung und Bedürfnisse der Haut des älteren Menschen ist dafür eine wesentliche Voraussetzung.

## Introduction and background

The demographic change has far-reaching consequences for our healthcare system, especially due to a change in the spectrum of diseases towards an increase in age-associated and chronic diseases [6]. Consequently, the number of people in need of long-term care is increasing. Currently, about 810,000 persons are residents of nursing homes (NHs) in Germany [20]. Providing appropriate care for people of advanced age is associated with particular challenges, especially for older people in NHs: residents tend to experience restrictions in activities of daily living, and frequently suffer from considerable cognitive and communicative deficits. Outpatient general practitioners (GPs) as well as outpatient specialist healthcare practitioners provide nursing home residents (NHRs) with healthcare services. Studies show that almost all residents regularly contact a GP [1, 17]. Inadequate care for NHRs is shown in dental as well as ophthalmological care [4, 21, 23]. There are only a few studies on dermatological care of older people, but national as well as international studies show that the care of the skin in older people is of special importance [5, 10, 11]. Skin ageing is related to anatomical and physiological changes associated with a reduced functional capacity of the skin, which increases its susceptibility to skin diseases and functional disorders [5]. At the same time, age-associated factors such as immobility and incontinence lead to vulnerable skin conditions, which mainly concerns NHRs [10]. Older NHRs often suffer from pruritic dry skin (xerosis cutis), fungal infections, (pressure) ulcers, dermatitis and skin tumors (benign and malignant) [5, 15].

Within this situation, the current research project aims to analyze the dermatological care of older psoriasis (PS) patients before and after entering an NH as well as predictors of dermatological care from claims data.

## Study design and investigation methods

The present analysis is based on data of the DAK-Gesundheit (DAK-G), a German statutory health insurance. The claims (routine) data cover a 40% representative sample (2.4 million) of all insured people of the DAK‑G on 31 December 2010. Data of these insured people are eligible for follow-up across all service areas through 31 December 2015. The statutory health insurance is essential within the German healthcare system, where about 90% of the German population (approximately 73 million people) is insured [7]. The data contain all billing relevant information from the outpatient and inpatient sector, including outpatient prescribed drugs based on the Anatomical Therapeutic Chemical (ATC) classification, coded diagnoses according to the German modification of the International Classification of Diseases (ICD-10-GM) and outpatient contacts with physicians at a quarter year level.

Every statutory health insurance fund also includes a statutory long-term care insurance fund. The data of the German long-term care insurance (*Gesetzliche Pflegeversicherung*) contains information about the date of nursing home admission (NHA) as well as the degree of care needed (ranging from care level 1: considerable need of care; to care level 3: most heavily care-dependent; level 4: hardship cases).

Our study includes all insured people of the DAK‑G sample aged 65 years and older, who were newly admitted to an NH between 1 January 2011 and 31 December 2014, with a subsequent insurance period in which the insured person has been insured for at least one day in each of the following four quarters. Also, they had to have received at least one confirmed ambulatory (outpatient) diagnosis of PS or at least one main diagnosis of PS on discharge from inpatient care in the year before NHA to be included in the study population. The ICD codes for identifying patients with PS (ICD-10 L40) were used (Table S1).

### Severe form of PS

For identifying patients with a severe form of PS, the following criteria were used:

- One inpatient main diagnosis of PS or
- at least one prescription for the drugs based on the ATC information within the corresponding year (Table S2).

For the present analysis, hardship cases (care level 4) were assigned to care level 3. The highest care level indicated the need for long-term care. The NHA quarter (index quarter) was assigned to the time before nursing home entry.

For analyzing the medical care before and after entering an NH with special attention to dermatological care, we examined at least one contact of a medical specialist (dermatologist, internist, neurologist/psychiatrist, urologist, gynecologist and ophthalmologist) or a GP. A stratified analysis was also performed to detect which group of specialists made the diagnosis of PS. The number of quarters in which the patient consulted a doctor for PS was compared between the year before vs. after NHA.

Specific comorbidities that play an important role in PS, in dermatology as well as in geriatric care, were evaluated. The following diagnoses according to the ICD-10 classification were examined: diabetes mellitus (E11, E12, E14), obesity (E66), hypertension (I10-I13), chronic ischemic heart disease (I25), osteoporosis (M80-M85), pruritus (L29), cataract (H25-H26), depression (F32, F33) and dementia (F00, F01, F02.0, F02.3, F03, G30, G31.0, G31.1, G31.82, G31.9) [2, 16]. The most frequent diagnoses made by dermatologists in the outpatient care sector [9] were also determined in the analyses (atopic dermatitis, other and unspecified dermatitis, skin cancer, screening procedures and diagnosis dermatophytosis, other superficial mycoses). Diagnoses were considered if they were coded at least once as a confirmed diagnosis in the outpatient setting in the year before or after NHA. The comorbidities were used to describe the burden of disease and were included as predictors in the multivariate analysis.

If at least one prescription was issued for one of the selected drugs within the year of observation, this is referred to as prescription prevalence. The following ATC information was used to analyze topical steroid treatment (Table S3).

As systemic steroids, the ATC information prescriptions with H02AB or H02B were included, for biological and nonbiological systemic agents see Table S2.

### Statistical analysis

The focus of the exploratory study was to report on medical care for PS patients one year before and after entering an NH, considering dermatological care in particular. We compared the proportion of PS patients with at least one specialist contact before and after NHA. The McNemar’s test was used to compare the two time periods [22]. Subsequently, factors were identified which may influence medical care by dermatologists after NHA. As independent variables, age groups in years (65–74, 75–84, 85+ years), sex (male vs. female), level of care (three levels), predefined diseases and prescriptions (at least one prescription for topical steroids, systemic steroids, biological/nonbiological systemic agents [Table S2, S3]) were included in the multivariable logistic regression model. Level of significance was set at 0.05. This was followed by a presentation of the proportions of diagnoses for psoriasis per coding medical specialty group in a before-after comparison as well as the number of diagnosis quarters for the diagnosis of psoriasis received from an insured person. We conducted all statistical analyses using SAS for Windows, Version 9.4 (SAS Institute Inc., Cary, NC, USA).

## Results

About 1200 insured people were newly admitted to an NH between 2011 and 2014 and were diagnosed with PS. Data were consistently available during the period of one year before to one year after entering the NH for 718 of these patients. In the year before entering the NH, the mean age of patients with PS was 83.3 (SD 7.5) years and they were predominantly female (76%). About 97% of patients with PS had a mild form of the disease throughout the study period (Table 1).

**Table 1:** Baseline characteristics of patients with psoriasis before and after entering a nursing home (*n* = 718)

	1 year before NHA ^a^, *n* (%)	1 year after NHA, *n* (%)
*Mean age (years) at NH entry (SD)*	83.3 (± 7.5)	
*Age groups, in years (%)*		
65–74	109 (15.2)	
75–84	251 (34.9)	
85+	358 (49.9)	
*Sex*		
Male	171 (23.8)	
Female	547 (76.2)	
*Care level*		
None/1	456 (63.5)	
2	231 (32.2)	
3	31 (4.3)	
*Type of diagnosis*		
Ambulatory care	712 (99.2)	453 (63.1)
Inpatient care	–	–
Both	6 (0.8)	5 (0.7)
*Severity of disease*		
Mild	694 (96.7)	699 (97.4)
Moderate to severe	24 (3.3)	19 (2.6)
*Prevalence of psoriasis-related geriatric comorbidities*		
Diabetes mellitus	277 (38.6)	258 (35.9)
Obesity	120 (16.7)	82 (11.4)
Hypertension	612 (85.2)	571 (79.5)
Ischemic heart disease	268 (37.3)	226 (31.5)
Osteoporosis	240 (33.4)	213 (29.7)
Depression	293 (40.8)	248 (34.5)
Cataract	214 (29.8)	184 (25.6)
Pruritus	51 (7.1)	64 (8.9)
Dementia	382 (53.2)	441 (61.4)
*Dermatological diagnosis*		
Atopic dermatitis	43 (6.0)	56 (13.4)
Other and unspecified dermatitis	193 (26.9)	225 (54.0)
Skin cancer	78 (10.9)	53 (17.7)
Skin cancer screening procedures and diagnosis	107 (14.9)	38 (5.3)
Dermatophytosis, other superficial mycoses	100 (13.9)	102 (24.5)
At least one dermatological diagnosis	349 (48.6)	334 (46.5)
*Pharmacotherapy*		
Nonbiological and biological systemic agents	19 (2.7)	15 (2.1)
Systemic steroids	126 (17.5)	102 (13.8)
Topical steroids (ATC: D07A)	245 (34.1)	252 (35.1)

*SD* standard deviation, *NHA* nursing home admission

^**a**^ including index quarter

Hypertension and dementia were among the most common disorders before and after NHA. The proportion of insured people with topical steroid therapy slightly increased in the same period (34% vs. 35%).

The first step was to analyze at least one medical contact of patients with PS, regardless of the reason, in comparison before and after NHA. The analyses showed that almost all NHR had contact with a GP during the observation period. The proportion of patients who had at least one contact with a specialist decreased after entering an NH, except for neurologists/psychiatrists and GPs. This downward trend in contacts was particularly noticeable among dermatologists and internal specialists (Table 2).

**Table 2:** Outpatient care of nursing home residents with psoriasis before and after nursing home admission (at least one contact per medical specialist group in the year before vs. after admission to a nursing home, *n* = 718)

Medical specialists	1 year before NHA ^a^, *n* (%)	1 year after NHA, *n* (%)	*P*-value
*General practitioner*	716 (99.7)	716 (99.7)	1.00
*Dermatologist*	320 (44.6)	288 (40.1)	**0.035**
*Internal specialist*	218 (30.4)	164 (22.8)	< 0.001
*Ophthalmologist*	281 (39.1)	254 (35.4)	0.068
*Neurologist/psychiatrist*	282 (39.3)	301 (41.9)	0.146
*Urologist*	157 (21.9)	139 (19.4)	0.086
*Gynecologist*	82 (11.4)	72 (10.0)	0.326

*NHA* nursing home admission

*P*-values in bold indicate statistical significance

^**a**^ including index quarter

Factors that impacted on the chance of having at least one dermatological contact after entering an NH were analyzed. The NHRs with PS who had already received dermatological care before NHA appeared to have the highest chance to contact a dermatologist after NHA, if controlled for all other variables (OR: 3.87; 95% CI: 2.70–5.54). Only topical therapy with steroids was shown as a significant predictor for dermatological care after NHA (OR: 1.61; 95% CI: 1.14–2.28) (Table 3).

**Table 3:** Multivariable logistic regression: predictors of dermatological care (at least one visit of a dermatologist in the year after nursing home admission)

Parameters	Reference	Odds ratio (95% CI)
*Age groups (years)*		
65–74	85+	1.29 (0.79–2.11)
75–84	85+	1.03 (0.71–1.50)
*Sex*		
Male	Female	0.89 (0.60–1.34)
*Care level*		
0/1	3	1.54 (0.67–3.53)
2	3	1.29 (0.55–3.01)
*Comorbidities*		
Dementia	No dementia	0.96 (0.69–1.34)
Pruritus	No pruritus	0.77 (0.41–1.45)
Cataract	No cataract	0.90 (0.63–1.29)
Depression	No depression	0.87 (0.62–1.22)
Diabetes mellitus	No diabetes mellitus	1.18 (0.83–1.67)
Obesity	No obesity	1.01 (0.64–1.59)
Ischemic heart disease	No ischemic heart disease	0.87 (0.61–1.22)
Osteoporosis	No osteoporosis	0.82 (0.57–1.19)
Hypertension	No hypertension	0.73 (0.46–1.16)
At least one other dermatological disease	No dermatological disease	1.24 (0.88–1.76)
*Pharmaceutical therapy*		
Biological/nonbiological systemic therapy	No systemic therapy	0.82 (0.29–2.30)
Systemic steroid therapy	No systemic steroid therapy	1.13 (0.73–1.76)
Topical steroids (D07A)	No topical steroids	**1.61 (1.14–2.28)**
*≥* *1 dermatological visit*	No dermatological visit	**3.87 (2.70–5.54)**

Values in bold indicate statistical significance

### Psoriasis-specific medical care

**Fig. 1:**
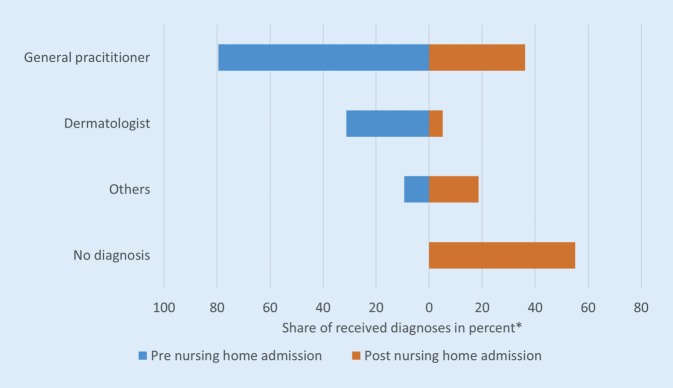
Share of coding specialist group of patients with psoriasis. *Asterisk* not additive as multiple answers possible

Before entering an NH, PS was diagnosed by a GP in 80% of patients, and in 30% by a dermatologist (multiple diagnoses possible) (Fig. 1).

More than 53% of the PS patients received at least 1 diagnosis of PS in all 4 quarters. Of those who received a diagnosis even after entering the NH, 69% receive at least 1 diagnosis in all 4 quarters.

## Discussion

Our study closes a gap and reports on the care of older PS patients before and after entering an NH. Due to a high frequency of somatic and cognitive comorbidities and often related multidrug therapy, management of older PS patients is challenging [8]. So far, there is little information on dermatological healthcare of NHR and, to our knowledge, no studies on the group of PS patients in NHs [11–13, 15].

### Medical contact

Our analysis shows that the portion of PS patients with dermatological contact, regardless of the reason, is significantly reduced after NHA compared to the percentage before entering the NH (45% vs. 40%). Research on the utilization of healthcare in older people in need of long–term care, especially with dermatological diseases, is limited. Compared to a recent study from Germany on medical specialist care before and after NHA of all NHR, the proportion of dermatological contacts in our analysis of PS patients is considerably higher. Only 16% vs. 18% according to NHA received dermatological care [19]. It appears that PS patients have a higher rate of dermatological contact than the general NH population. This higher contact rate is clinically understandable to infer. Our results are consistent with the findings of another German health insurance data-based study, where 54% of NHRs with a dermatological disease and a low care level did not have dermatological specialist contact [18].

The reasons for the reduced dermatological contacts can be manifold and would require further research: It has to be discussed whether dermatological care gets out of focus in the context of more life-threatening or severe acute diseases. Another approach would be that older patients show a milder clinical picture and further dermatological treatment is not necessary. A further explanation could be that treatment initiation by a dermatologist leads to improvement of the condition which enables further care by a GP.

It is therefore important that both the medical as well as the nursing staff keep a watchful eye on these symptoms and drug use in the context of PS. Adequate training and continuing education can therefore lead to an appropriate skincare. The finding that NHRs are to a large extent cared for by a GP is well documented, both in the current study and in the literature [1, 17, 19]. Overall, the results must be read in the context of a scarcity of specialists (6200 dermatologists are currently active compared to about 42,200 GPs plus internal medicine GPs [3]). Only a few dermatologists make visits to NHs; at the same time, NHR have significantly limited mobility, and their often reduced cognitive abilities also make medical visits outside the nursing home difficult. Hence, especially in NHs, the GP acts as a central hub that can identify and channel health-related needs. This therefore requires geriatric and gerontodermatological expertise, especially from the GP.

The strongest predictor for a dermatological contact after admission to an NH was dermatological contact before NHA. This finding is also consistent with the named study of Ramos et al. regarding rheumatological care [14]. Apparently, the time before NHA already determines the dermatological care (at least one dermatological contact) in the NH. Surprisingly, age, sex, care level and selected diseases showed no effect in the present analysis. Only the prescription of topical steroids could be identified as another significant factor. The dermatological supervision in the prescription of topical preparations for steroids is desirable.

The diagnosis of PS is mainly made by GPs and in about 30% by dermatologists in the year prior to NHA. It must be discussed whether a dermatologist contact is not necessarily due to an inactive phase of the disease or whether this is representing an undersupply. These analyses also show the special position of the GPs in the context of care for people of advanced age with PS.

## Strengths and limitations

The results must be interpreted in the context of the strengths and limitations of the present study. We were able to compare a large cohort of newly admitted NHRs with PS diagnoses in the year before NHA. By using the health insurance funds data, we were able to include a cohort irrespective of very old age, frailty, institutionalization or cognitive impairment. At the same time, the use of this database also means restrictions: there are no clinical parameters that could for example provide information on the severity and activity of the disease. Due to the data structure of ambulatory medical care, diagnoses are only available quarterly. We have decided to include the quarter of NHA in the period before admission to ensure that all insured people also live in the nursing home during the second year of examination. Analyses do not include insured people with PS who did not survive at least one year after NHA (*n* = 430), as the focus of the current analysis is on assessing healthcare for a chronic condition. The closer the time to the insured’s death, the more likely life-threatening conditions become the center of attention for healthcare. It was therefore important to the researchers to study a sufficiently long and comparable time at risk for examining the healthcare situation of a chronic but primarily nonfatal disease. The results of the routine data analysis were discussed in a second, qualitative part of the project (focus groups, interviews) with nurses, nurse managers, dermatologists and primary care physicians. As this is an exploratory study, the results need to be investigated in further studies.

## Conclusion

The analysis of health insurance data showed that the number of PS patients with dermatological contact in the year before NHA significantly decreased after entering the NH. The strongest predictor for a dermatological contact after NHA is the presence of dermatological contact before NHA. The course of the dermatological care in the NH seems to be set already before entry into the NH. Consequently, special attention must be paid to skin health in the NH to counteract an undersupply. Specific dermatological knowledge of the skin of the older people is therefore required from all those involved in the care process.

## Practical conclusion

- Time before entering a nursing home seems to be an important marker for healthcare continued in nursing homes.
- Analyses reinforce the central position of the GP.
- Dermatological knowledge of the skin of the older people is highly relevant for all those involved in the provision of healthcare services.

## Supplementary Information

**Table S1** Psoriasis diagnosis (ICD-10-GM codes). **Table S2** Substances indicating a severe form of psoriasis (approval before 2016). **Table S3** Topical steroid therapies according to Anatomical Therapeutic Chemical (ATC) information

## Declarations

The study was conducted according to the Declaration of Helsinki. We considered the criteria of the national good practice guidelines. According to the Good Practice of Secondary Data Analysis, a national guideline for the use of administrative databases, no approval of an ethical committee was required.
