# Supplementary material for: Health services in older psoriasis patients before and after nursing home admission: A retrospective analysis of health insurance data
Source: Z Gerontol Geriatr. 2022 Mar 21;56(2):139–45. doi: 10.1007/s00391-022-02020-y (PMC10011290; doi:10.1007/s00391-022-02020-y)
Supplement: Supplementary file 1 — Table S1 Psoriasis diagnosis (ICD-10-GM codes). Table S2 Substances indicating a severe form of psoriasis (approval before 2016). Table S3 Topical steroid therapies according to Anatomical Therapeutic Chemical (ATC) information [file 391_2022_2020_MOESM1_ESM.docx]

**Health Services in Residents with Psoriasis – Before and After Nursing Home Admission, Supplement**

**Table S1** Psoriasis diagnosis (ICD-10-GM codes)

| ICD-10 diagnosis | Term |
| --- | --- |
| L40.0 | Psoriasis vulgaris |
| L40.1 | Generalised pustular psoriasis |
| L40.2 | Acrodermatitis continua |
| L40.3 | Pustulosis palmaris et plantaris |
| L40.4 | Guttate psoriasis |
| L40.5 | Arthropathic psoriasis |
| L40.8 | Psoriasis, unspecified |
| L40.9 | Other psoriasis |

**Table S2** Substances indicating a severe form of psoriasis (approval before 2015)

| ATC information | Active ingredients |
| --- | --- |
| Biological systemic agent | |
| L04AB04 | Adalimumab |
| L04AB02 | Infliximab |
| L04AC05 | Ustekinumab |
| L04AC10 | Secukinumab |
| L04AB01 | Etanercept |
| Nonbiological systemic agent | |
| L04AA32 | Apremilast |
| L01BA01 | Methotrexate |
| M01CX01 | Methotrexate |
| L04AX03 | Methotrexate |
| L04AD01 | Cyclosporine |
| D05BB02 | Acitretin |
| D05BX20 | Alkyl esters of fumaric acid |
| D05BX01 | Fumaric acid |
| D05BX51 | Fumaric acid derivatives, combinations |

ATC, Anatomical Therapeutic Chemical

**Table S3** Topical steroid therapies according to Anatomical Therapeutic Chemical (ATC) information

| ATC information | Active ingredients |
| --- | --- |
| D07A | **Corticosteroids, plain** |
| D07AA | [Corticosteroids, weak (group I)](https://www.whocc.no/atc_ddd_index/?code=D07AA&showdescription=yes) |
| D07AB | [Corticosteroids, moderately potent (group II)](https://www.whocc.no/atc_ddd_index/?code=D07AB&showdescription=yes) |
| D07AC | [Corticosteroids, potent (group III)](https://www.whocc.no/atc_ddd_index/?code=D07AC&showdescription=yes) |
| D07AD | [Corticosteroids, very potent (group IV)](https://www.whocc.no/atc_ddd_index/?code=D07AD&showdescription=yes) |
